# Domestic dogs as reservoirs for African trypanosomiasis in Mambwe district, eastern Zambia

**DOI:** 10.1038/s41598-024-69834-1

**Published:** 2024-09-10

**Authors:** Malimba Lisulo, Boniface Namangala, Cornelius Mweempwa, Maxwell Banda, Herman Chambaro, Ladslav Moonga, Hayashida Kyoko, Sugimoto Chihiro, Kim Picozzi, Sutherland K. Maciver, Ewan T. MacLeod

**Affiliations:** 1 Infection Medicine, Deanery of Biomedical Sciences, College of Medicine and Veterinary Medicine, The University of Edinburgh, 1 George Square, Edinburgh, EH8 9JZ UK; 2 Central Veterinary Research Institute, Ministry of Fisheries and Livestock, P.O. Box 33780, Lusaka, Zambia; 3 Department of Paraclinical Studies, School of Veterinary Medicine, University of Zambia, P.O. Box 32379, Lusaka, Zambia; 4 Department of Veterinary Services, Tsetse and Trypanosomiasis Control Section, Ministry of Fisheries and Livestock, Lusaka, Zambia; 5 Research Centre for Zoonosis Control, Hokkaido University, Kita-Ku, Sapporo, 001-0020 Japan; 6 Centre for Discovery Brain Sciences, Edinburgh Medical School, University of Edinburgh, Edinburgh, EH8 9XD UK

**Keywords:** AT, HAT, Dogs, Reservoir, Trypanosomes, Mambwe district, Zambia, Parasite host response, Infectious-disease diagnostics

## Abstract

The control of African trypanosomiasis (AT) in Eastern and Southern Africa, including Zambia, faces huge challenges due to the involvement of wild and domestic animal reservoirs. Free-roaming dogs in wildlife-populated and tsetse-infested villages of Zambia’s Mambwe district are exposed to infectious tsetse bites. Consuming fresh raw game meat and bones further exacerbates their risk of contracting AT. We focus on the reservoir role of such dogs in maintaining and transmitting diverse species of trypanosomes that are infective to humans and livestock in Zambia’s Mambwe district. A cohort of 162 dogs was enrolled for follow-up at 3 different time points from June to December 2018 in selected villages of Malama, Mnkhanya, and Nsefu chiefdoms of Mambwe district, eastern Zambia. Blood and serum were screened for AT by microscopy, GM6 ELISA, PCR (ITS1 and SRA), and Sanger sequencing. Out of the 162 dogs in the cohort, 40 were lost to follow-up and only 122 remained traceable at the end of the study. GM6 ELISA detected *Trypanosoma* antibodies in 121 dogs (74.7%) and ITS1-PCR detected DNA involving single and mixed infections of *T. congolense*, *T. brucei,* and suspected *T. simiae* or *T. godfreyi* in 115 dogs (70.9%). The human-infective *T. b. rhodesiense* was detected by SRA PCR in 67 dogs (41.4%), and some sequence data that support the findings of this study have been deposited in the GenBank under accession numbers OL961811, OL961812, and OL961813. Our study demonstrates that the *Trypanosoma* reservoir community in Zambia is wider than was thought and includes domesticated dogs. As dogs are active carriers of human and livestock-infective trypanosomes, they pose a risk of transmitting AT in endemic villages of Mambwe district as they are neglected and left untreated. To fully bring AT under control, countries such as Zambia where the role of animal reservoirs is important, should not limit their prevention and treatment efforts to livestock (especially cattle) but also include dogs that play an integral part in most rural communities.

## Introduction

African trypanosomiasis (AT), also known as sleeping sickness in humans or nagana in animals, is a devasting vector-borne disease affecting several sub-Saharan African countries including Zambia^1^. Different species of protozoan haemoflagellates (trypanosomes, genus *Trypanosoma*), produce a complex and fatal disease in humans and domestic animals if left untreated^2,3^. Trypanosomes are transmitted primarily through the bite of infected tsetse flies (genus *Glossina*) and to a lesser extent by other blood-feeding arthropods such as Tabanids (Horseflies) and Stomoxys (Stable flies)^4^. Communities that practice agriculture, animal husbandry, fishing, and hunting in tsetse-infested rural areas experience the devastating effects of AT with morbidities and mortalities mostly in livestock^5^.

Two different species of trypanosomes cause human AT (HAT): *Trypanosoma brucei gambiense* (endemic in Western and Central Africa) and *Trypanosoma brucei rhodesiense* (in east and southern Africa). *T. b. gambiens*e triggers a chronic human-tsetse-human transmitted disease that accounts for about 98% of all the reported HAT cases^6^. Humans are the major reservoirs of *T. b. gambiens*e infections, but domestic animals (i.e., pigs, small ruminants, and dogs) might also be involved in disease transmission^7^. *T. b. rhodesiense* on the other hand triggers an acute animal-tsetse-human transmitted disease that accounts for about 2% of HAT cases^6^. In most *T. b. rhodesiense* endemic countries, wildlife is the main sylvatic reservoir, and livestock is the domestic reservoir^8–12^.

Unlike in Western and Central Africa where humans are the main reservoirs of HAT, animal reservoirs (both wild and domestic) make controlling *T. b. rhodesiense* HAT in Eastern and Southern Africa hugely challenging. Zambia is one of the endemic countries facing this problem. Since 1908 when the first case of *T. b. rhodesiense* HAT was reported, sporadic cases believed to be spillover infections from wildlife continue to affect humans^13,14^. These cases are common in wildlife-domestic interfaces, particularly in the Luangwa and Zambezi Valley Basins which are historic foci of *T. b. rhodesiense* HAT in Zambia^10^.

Tsetse flies circulate various trypanosomes in Zambia during bloodmeal acquisition^15,16^ from wildlife “the sylvatic reservoirs”^17–19^ to livestock “the domestic reservoirs”^20,21^, and ultimately to humans^22–25^. Analysis of tsetse blood meals demonstrates that domesticated animals and humans act as alternative sources of food when preferred wildlife is scarce^26–30^.

The Luangwa Valley is the most active foci of AT in Zambia, particularly in the Eastern Province where cases are frequently reported. Most strains of trypanosomes have already developed resistance to the available treatment of diminazene aceturate and isometamidium chloride^31–34^. This is worrying especially when the human infective *T. b. rhodesiense* is involved. Other than being found in humans, the zoonotic *T. b. rhodesiense* in Zambia has also been detected in tsetse flies^35^, a wide range of wildlife species^18,19^, cattle^36,37^, and dogs^38,39^.

We had previously reported the presence of animal-infective *T. congolense*, *T. b. brucei*, and the human-infective *T. b. rhodesiense* in indigenous dogs of Mambwe district, eastern Zambia^39^. This initial cross-sectional study of 2012 by Lisulo et al.^39^, provided baseline data on the prevalence of canine AT in village dogs of Malama, Mnkhanya, and Nsefu chiefdoms of Mambwe district. We returned to the same area 6 years later in 2018 to assess the disease by enrolling a cohort of village dogs and repeatedly examining them over three seasons between June and December 2018 to detect health and demographic changes. Our most recent observations of free-roaming dogs in wildlife-populated and tsetse-infested villages of Mambwe district confirmed the occurrence of various helminths, tick-borne diseases, and AT^40^. Despite their importance for security and hunting, the dogs in this area were visibly malnourished, infested with ticks, excreted live adult tapeworms and roundworms in their stool, and generally lacked basic home and veterinary care. Moreover, most dogs had regular exposure to tsetse bites and consumption of fresh raw game meat and bones^40^, putting them at greater risk of acquiring AT through infected tsetse bites^41^ and oral lacerations as demonstrated elsewhere^42,43^.

In the current research, we focus on the reservoir role of such dogs in transmitting AT and explore the diversity of trypanosomes in the canine population of Mambwe district. We also attempt to use a non-commercial ELISA assay to detect *Trypanosoma* antibodies in naturally infected dogs. The assay uses GM6 antigen, a conserved repeat motif of 68 amino acids linked to the flagellum of *T. brucei* subspecies, *T. congolense*, and *T. vivax*^44^. So far, the GM6 antigen-based ELISA has been used to detect antibodies in cattle^44^, goats and sheep^45^, camels^46^, donkeys^47^, horses^48^, and water buffalos^49^.

## Results

**Figure 1:**
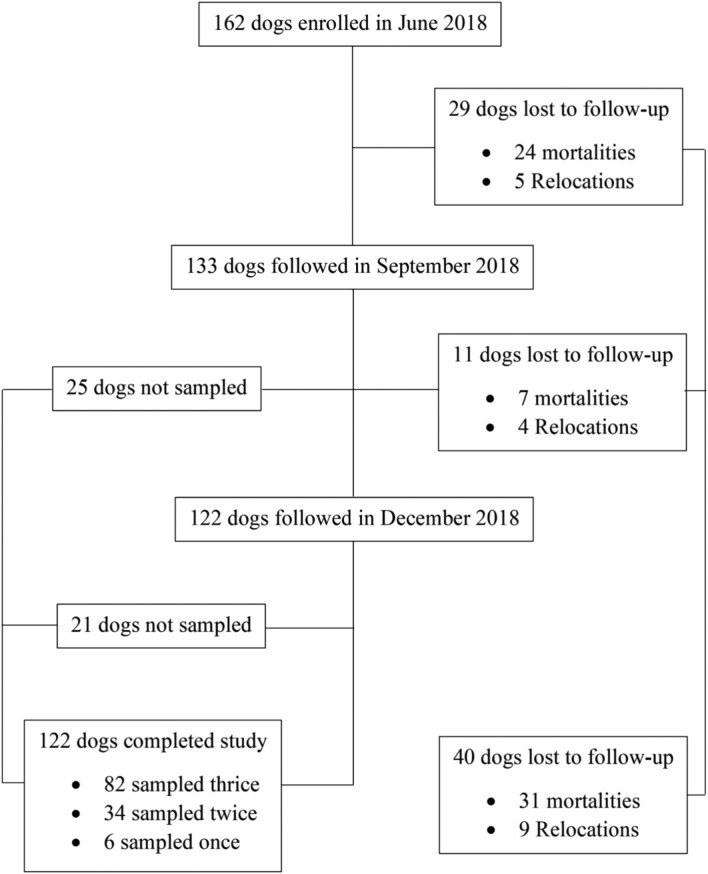
Flow chart showing follow-up of dogs in the cohort from June to December 2018.

Out of the 162 dogs in the cohort, 40 were lost to follow-up and only 122 remained traceable at the end of the study (June to December 2018). Dogs were reexamined based on availability at each subsequent follow-up (Fig. 1).

**Figure 2:**
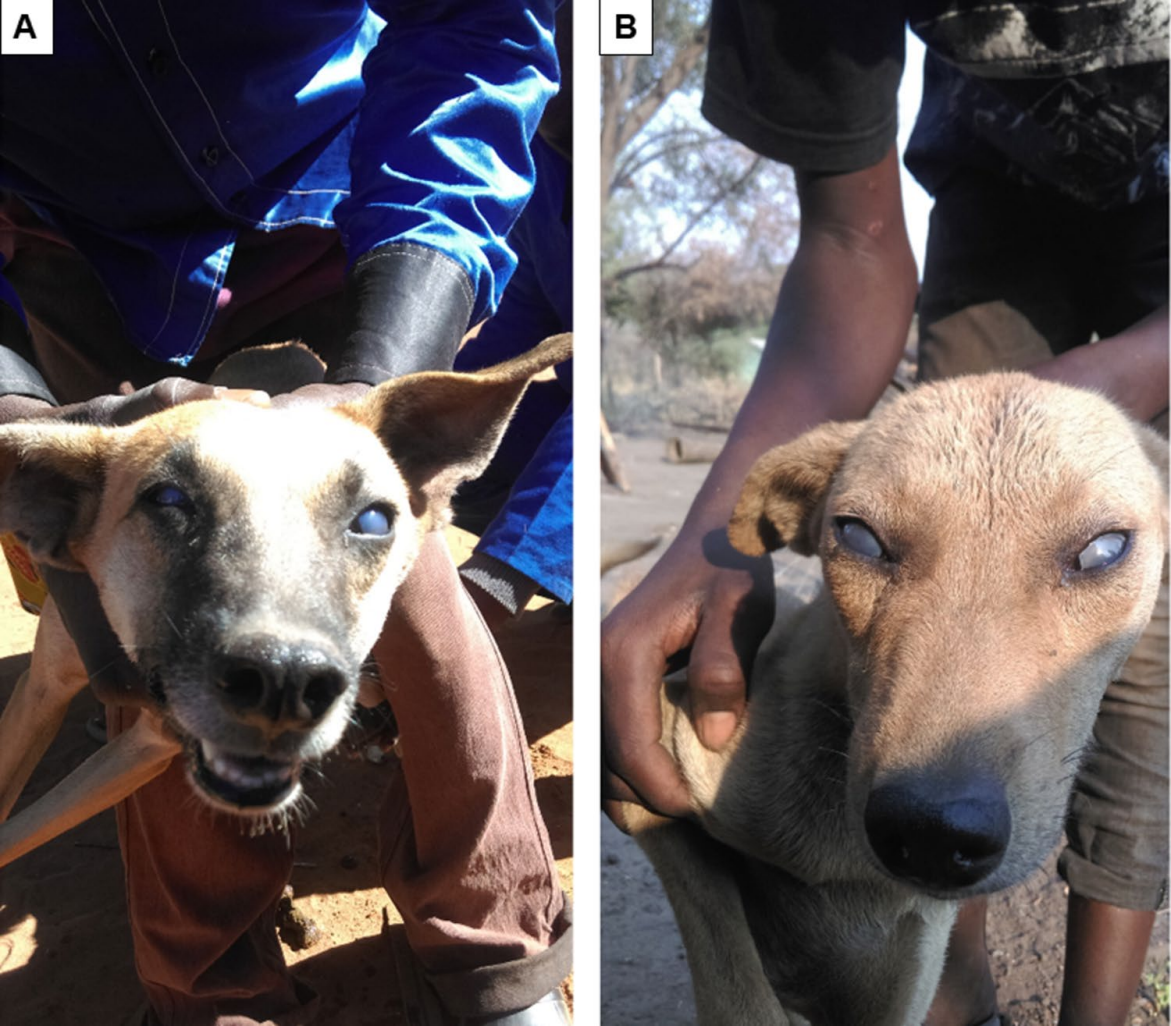
Corneal opacity in some dogs in the cohort. (**a**) Bluish eyes, and (**b**) greyish eyes.

Overall, 11 dogs (6.8%; 95% CI 3.8–11.8) developed corneal opacity (Fig. 2) and almost all were euthanized by their owners before treatment with diminazine aceturate could be initiated. Sixty dogs (37%; 95% CI 29.9–44.7) had temperature readings ≥ 39.5℃ (indicative of fever) and 105 dogs (64.8%; 95% CI 57.2–71.8) had anemia that ranged from mild (42%), to moderate (20.4%) to severe (2.4%) forms. Parasitic infections, injuries mainly caused by wildlife attacks, intestinal worms, and ectoparasites, were among the primary causes of canine morbidity and mortality.

### Detection by microscopy

**Figure 3:**
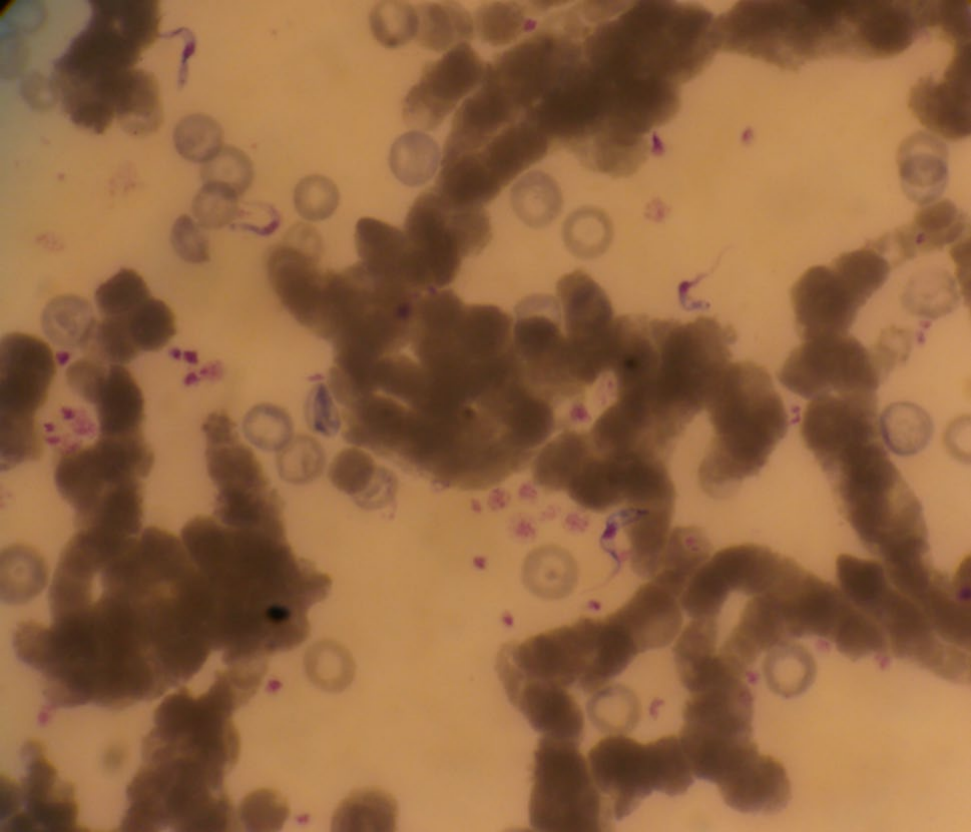
Dog trypanosomes propagating in mice. Giemsa-stained thin blood smear (× 1000).

Microscopy was conducted on all dogs at the first time point in June. The prevalence of trypanosomes in the blood was (8.0%; 95% CI 4.8–13.2). In most cases the parasitaemia was low, but when blood was injected into mice the parasitaemia increased several days post-inoculation (Fig. 3).

### Detection by ELISA

The GM6 ELISA detected *Trypanosoma* antibodies in 121 dogs (74.7%; 95% CI 67.5–80.8) at a calculated cut-off value of 0.215. The cohort had a mean OD value of 0.372 that ranged from 0.028 to 2.414. Seroprevalence in the cohort varied during follow-up with more cases detected in the rainy season (Table 1).

**Table 1:** Summary of GM6 ELISA and ITS1-PCR prevalence based on seasons.

Season (month)	No. of dogs tested	Seroprevalence (%)*	ITS1-PCR Prevalence (%)^φ^
Cold (June)	162	54.9	52.5
Hot (September)	106	63.9	43.4
Rainy (December)	101	69.3	62.4
Overall	162	74.7	70.9

*Seroprevalence included dogs that tested positive once (n = 49), twice (n = 38), and thrice (n = 34).

^φ^Prevalence included dogs that were positive once (n = 56), twice (n = 39) and thrice (n = 20).

### Detection by ITS1-PCR

**Figure 4:**
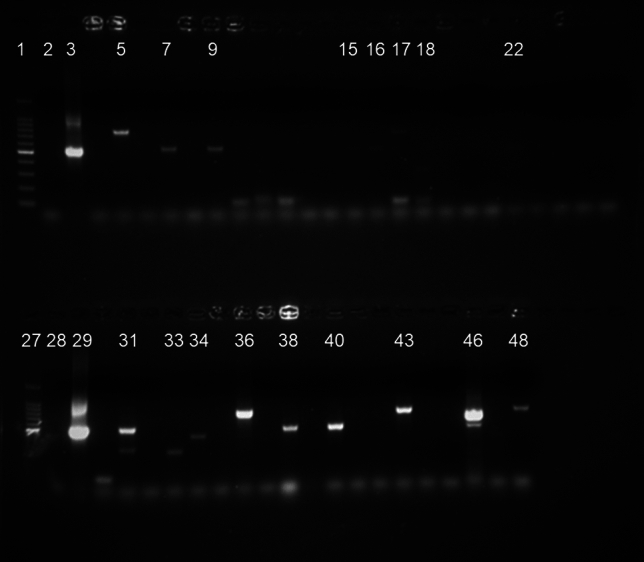
Gel electrophoresis of ITS1-PCR products on 1.5% agarose gel. Lanes 1, 27: marker; lanes 2, 28: negative control (water); lanes 3, 29: positive control (*Trypanozoon*); lanes 5, 17, 36, 43, 46, 48: *T. congolense*; lanes 7, 9, 15, 16, 17, 22, 31, 38, 40, 46: *T. brucei*; lanes 18, 31, 33, 34, 47: suspected *T. simiae* or *T. godfreyi*.

ITS1-PCR detected DNA involving single and mixed infections of *T. congolense*, *T. brucei,* and suspected *T. simiae* or *T. godfreyi* (Fig. 4) in 115 dogs (70.9%; 95% CI 63.6–77.4). Prevalence in the cohort varied during follow-up with more cases detected in the rainy season (Table 2). Of these dogs, 4 (2.5%; 95% CI 0.9–6.2) were positive at enrolment but remained negative at the two subsequent follow-ups.

**Table 2:** Prevalence of single and mixed trypanosomes in the cohort.

Species	Cold season (n = 162)		Hot season (n = 106)		Rainy season (n = 101)		Cumulative (n = 162)	
	%	95% CI	%	95% CI	%	95% CI	%	95% CI
T. c	6.2	3.4–11	6.6	3.2–13	7.9	4.1–14.9	14.2	9.6–20.4
T. b	27.8	21.5–35.1	29.2	21.4–38.5	31.7	23.4–41.3	46.3	38.8–54
T. b & T. c	4.3	2.1–8.7	7.5	3.9–14.2	19.8	13.2–28.6	17.3	12.2–23.9
T. b & T. c/T. s/T. g	8.0	4.8–13.2	0.0	0.0–3.5	2.0	0.5–6.9	9.3	5.7–14.7
T. s/T. g	6.2	3.4–11	0.0	0.0–3.5	1.0	0.2–5.4	7.4	4.3–12.5
Overall	52.5	44.8–60	43.4	34.4–52.9	62.4	52.6–71.2	70.9	63.6–77.4

T. c (*T. congolense*), T. b (*T. brucei*), T. s (*T. simiae*), T. g (*T. godfreyi*), % (prevalence), CI (Confidence Interval).

### Correlation among diagnostic tests

The 13 cases detected by microscopy were all accurately detected and confirmed that both tests were 100% sensitive: ELISA (kappa 0.134) and ITS1-PCR (kappa 0.146). However, there was only a slight agreement of 59.3% in case detection between ELISA and ITS1-PCR with a Kappa value of − 0.028. ELISA detected 36 positives that had no DNA and did not detect 30 samples with DNA (Table 3).

**Table 3:** Detection accuracy of GM6 ELISA and ITS1-PCR.

GMG ELISA	ITS1-PCR		Total
	Positive	Negative	
Positive	85	36	121
Negative	30	11	41
Total	115	47	162

Kappa − 0.028, sensitivity 73.9%, specificity 23.4%, positive predictive value 70.2%, negative predictive value 26.8%.

Generally, the number of trypanosome-positive dogs in the cohort increased with time. Both ELISA and PCR detected more cases at the onset of the rainy season in December. Although trypanosomes were detected in dogs in all chiefdoms, cases were most prevalent in Malama. Of the 11 cases with corneal opacity, 7 (63.6%) were positive by ELISA and 9 (81.8%) by PCR. The two undetected cases were euthanized before subsequent follow-up.

### Detection and sequencing of SRA gene

**Figure 5:**
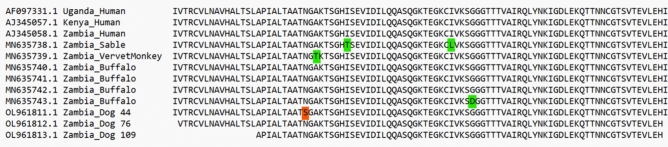
Alignment of our dogs’ SRA amino acid sequences with several GenBank references. Highlighted letters show the positions of alterations in the sequences.

The human-infective *T. b. rhodesiense* was detected in 67 dogs (41.4%), which included dogs that tested positive once (n = 53), twice (n = 12), and thrice (n = 2). Some sequence data that support the findings of this study have been deposited in the GenBank under accession numbers OL961811 (dog 44, Nsefu chiefdom), OL961812, and OL961813 (dogs 76 and 109, Mnkhanya chiefdom, respectively). These SRA nucleotide sequences were 99.7% (dog 44) and 100% (dogs 76 and 109) identical to SRA reference genes. Dog 44 had a unique alteration in its amino acid sequence, asparagine was replaced by a serine thereby distinguishing it from known references (Fig. 5).

## Discussion

Our study confirms that the reservoir community of trypanosomes in Zambia is wide and includes man’s best friend, “dogs”. Scanty trypanosomes or low parasitaemia suggest that most dogs had chronic infections. This is perhaps why some dogs recorded persistent infections throughout the 6-month follow-up period, long enough for infection to be passed on to other hosts. The propagation of scanty trypanosomes in mice indirectly implies that in the presence of competent arthropod vectors such as tsetse flies, tabanids and stomoxys flies which are abundant in Mambwe district, canine infections could be transmitted to wildlife, livestock, and humans.

The occurrence of single and mixed infections of *T. congolense*, *T. brucei,* and *T. b. rhodesiense* is concerning given that the former and latter cause devastating diseases in cattle and humans, respectively. However, the presence of these trypanosomes in dogs is not coincidental here, they have previously been reported in these chiefdoms^39^. More attention needs to be given to dogs in this area by relevant authorities. The detection of the SRA gene in trypanosomes isolated from many dogs poses a public health threat considering the close relationship between humans and dogs. Our research agrees with^41^ who also amplified the SRA gene from dogs in Zambia. Other potential reservoirs in Zambia where the SRA gene has been amplified include cattle^36,37^, and wildlife (i.e., buffalo, vervet monkey, sable)^18,19^. The SRA gene has also been amplified from tsetse flies^35^, and humans^50,51^ in Zambia. Whereas detecting “suspected” *T. simiae* and *T. godfreyi* positive cases is a new and interesting finding in dogs, our study did not verify these cases by sequencing but on the size of ITS bands on agarose gels.

Accurate diagnosis is therefore critical in detecting and preventing AT in endemic rural areas. However, most rural health facilities do not have modern sensitive tests and equipment to perform ELISA or PCR other than low-sensitive microscopy. Using GM6 ELISA (74.7%) and ITS1-PCR (70.4%), we were able to demonstrate in this study that microscopy (8%) was about 9 times less sensitive in detecting cases with kappa values of 0.134 and 0.146, respectively. The fact that the two tests picked up more positives than microscopy suggests that parasitaemia was below detectable levels on a microscope. Although we have compared GM6 ELISA and ITS1-PCR, we also want to point out that the two tests do not target the same thing. The former detects antibody titres that show exposure rather than potential active infections detected by the latter. Therefore, recent infections might mean no antibody detection or vice versa, so the two tests might show slight agreement in detecting cases as observed in our current findings. As this was the first attempt at using GM6 ELISA to detect AT seroconversion in naturally infected dogs, more research is needed to optimize the protocol. Otherwise, GM6 ELISA is a cheaper alternative diagnostic tool to ITS1-PCR that can be utilized by AT endemic facilities that rely on microscopy. Moreover, dogs in AT-endemic areas such as Mambwe district are neglected and do not receive treatment when infected or injured^40^.

In the absence of diagnostic tools, dog owners living in tsetse-infested areas such as Mambwe district can use corneal opacity as a sign to suspect canine AT. Corneal opacity is a common clinical feature of canine AT associated with *T. brucei*-subspecies infections^41,52–57^. Amongst the pathognomonic clinical features of canine AT, corneal opacity was found to be one of the reliable predictors that can be used by dog owners in Mambwe district to suggest the involvement of trypanosomes^39^. By ELISA and PCR, we found 63.6% and 81.8% positivity amongst dogs with corneal opacity. The 11 cases of corneal opacity recorded in the current study mostly developed at the onset of the hot season from August onwards when cases were seen to increase. This corresponds with the influx of tsetse flies around human settlements “villages” in the hot season. Whereas infections could be due to infectious tsetse bites, the risk of AT in these dogs is further exacerbated by consuming fresh raw game meat and bones that can be acquired through oral lacerations^40^.

Our study demonstrates that the *Trypanosoma* reservoir community is wide and dogs that play an integral part in human establishments are active carriers. Humans and other mammalian hosts living close by are at risk as dogs are commonly left untreated. To fully bring AT under control, countries such as Zambia where the role of animal reservoirs is important, should not limit their prevention and treatment efforts to livestock (especially cattle) but include dogs. We are also cognisant of the fact that Mambwe district is not the only region in Zambia impacted by AT and tsetse flies, so it would be beneficial for other studies to investigate the current state of canine trypanosomes in other affected areas. Future research should compare the prevalence of trypanosomes in dogs owned by new settlers to those owned by long-term residents, as new settlers are more likely to live in areas with higher tsetse fly infestations as is the case in Mambwe district. Moreover, as our study was limited in the duration of follow-up, we recommend that future studies should examine dogs for a much longer period to generate meaningful inferences on seasonal data.

## Methods

### Study area

Mambwe District, located in the Eastern Province of Zambia, spans approximately 5724.5 km^2^ and has a human population of 119,313, with a density of 20.8 persons per km^2^^40^. The canine population is estimated at 3155 (according to the Department of Veterinary Services, Mambwe District). Situated within the tsetse-infested Luangwa Valley basin, a historical hotspot for African trypanosomiasis (AT), the district’s altitude can drop as low as 300 m above sea level. Mambwe experiences three seasons: a cold-dry season from May to early August, a hot-dry season from mid-August to mid-November with temperatures ranging from 32 to 43 °C, and a rainy-hot season from mid-November to April. The district is prone to localized flooding due to its location, with rainwater from higher neighbouring districts draining into Mambwe. Prolonged rainfall can overwhelm local drainage systems, rivers, and streams, causing flooding in low-lying areas and rendering most areas inaccessible by road except by boat or helicopter from November to May.

Mambwe District is rich in wildlife. Its game management area (GMA) serves as an extension of the South Luangwa National Park (SLNP), hosting a variety of wildlife, including mammals, reptiles, and birds that migrate in and out of the district^58^. This study was conducted in wildlife-populated and tsetse-infested villages of Malama, Mnkhanya, and Nsefu chiefdoms in Mambwe district. The three chiefdoms were selected to encompass a variety of natural and human habitats and on previous cases of canine AT^39^.

i. Malama Chiefdom is situated in a densely wildlife-populated area on the outskirts of the SLNP. This remote location is heavily infested with tsetse flies and at the time of sampling had almost no livestock, except fowls and dogs. With the recent influx of new settlers, the livestock situation is more likely to change.
ii. Mnkhanya Chiefdom is located on the edge of a wildlife zone, far from the SLNP. It has a very mild presence of wildlife and tsetse flies, with livestock and dogs being common.
iii. Nsefu Chiefdom lies within a moderately wildlife-populated area. It has a moderate tsetse infestation, and livestock and dogs are prevalent.

### Selection criteria

Building on the findings of Lisulo et al.^39^ regarding the presence of AT in indigenous dogs in the Malama, Mnkhanya, and Nsefu chiefdoms of Mambwe district, we extended our current study to every dog-keeping village within these chiefdoms. Participation was voluntary, and only villages where dog keepers expressed a willingness to participate were included in the study. Individuals who did not consent or were below the minimum age of 12 were excluded. Consequently, we focused on 58 dog-keeping villages that expressed a willingness to participate: Malama (15 villages), Mnkhanya (18 villages), and Nsefu (25 villages). These included villages with previously identified cases of AT in dogs and additional villages that kept dogs. The selected villages were typically small, comprising one or more households from the same family. All three chiefdoms are predominantly inhabited by Kunda-speaking people, with other Zambian tribes as minority settlers. We enrolled male and female dogs aged over 3 months as part of the cohort. Although puppies as young as six weeks have been used in Trypanosoma in vivo studies elsewhere^55^, we excluded puppies less than 4 months old because they could still carry maternal antibodies against trypanosomes, which was one of the things we tested for in this study. No additional households or dogs were included after the initial enrolment, and puppies born to females in the cohort were not considered^40^.

### Study design

A cohort of 162 indigenous dogs (82 males and 80 females) from 93 dog-keeping households were enrolled for follow-up. On average, each household had 1.7 dogs (± 1.1), with the number of dogs per household ranging from one to five. The cohort’s median age was 30 months (± 25), with ages spanning from four to 156 months. The dogs, from Malama (31 dogs), Mnkhanya (68 dogs), and Nsefu (63 dogs) were repeatedly examined for pathogens, injuries, illnesses, and mortalities. The cohort of dogs was monitored for 6 months, from June to December 2018, to track changes in their health and demographics. Throughout this period, the dogs were visited at three distinct time points, corresponding to different seasons: the initial visit and enrollment took place in June (cold-dry season), the second visit occurred in September (hot-dry season), and the final visit was in December (rainy-hot season)^40^.

As an incentive to the dog owners, each dog in the cohort was freely vaccinated against rabies at enrollment, dewormed at the second or third visit with intramuscular ivermectin (0.5 ml/25 kg body weight), and where necessary treatment was provided for injured dogs (i.e., wounds were cleaned and antiseptics applied) and those confirmed with motile trypanosomes were administered with 0.1 ml REDNIL per 2 kg live mass (Ready-to-use injectable solution containing diminazene aceturate 70 mg/ml, Phenazone 375 mg/ml). Our study did not involve the use of anaesthesia or euthanasia.

### On-site activities

#### Physical examinations

During each visit, trained district veterinary staff based at Kakumbi Tsetse and Trypanosomiasis Research Station (KTTRS) in Mambwe district physically examined the dogs for abnormalities, injuries, and ectoparasites, scored the general body conditions, and measured anal temperatures. Therefore, any clinical change in the cohort such as fever, emaciation, pallor of visible mucous membranes, enlarged superficial lymph nodes, general oedema, corneal opacity, and other related clinical features was recorded.

#### Blood sampling

About 2 ml of whole blood was collected from the cephalic vein of each dog using sterile vacutainer needles and tubes (Plain and EDTA tubes).

#### Microhematocrit centrifugation technique

Blood in EDTA tubes was drawn into heparinized capillary tubes, sealed with plasticine, and centrifuged for 5 min at 5000 rpm using an on-site petrol-powered generator. Packed cell volume (PCV) values were read using a haematocrit reader, with values below 37% indicating anaemia, categorized as mild (30–36%), moderate (15–29%), or severe (< 15%)^59^.

#### Buffy coat examination

The buffy coats and upper layers of red blood cells were placed on glass slides, covered with coverslips, and examined on-site. Trypanosomes were identified by their motility characteristics: *T. brucei* (varied sizes, rapid movement with undulating membrane creating light pockets), *T. congolense* (small, sluggish, adheres to red blood cells), *T. vivax* (large, very active, quickly crossing the field with occasional pauses), and *T. theileri* (large, tends to rotate or stay immobile with whip-like movements)^60^. If trypanosomes were present, thin Giemsa-stained smears were prepared from blood in EDTA for detailed analysis. Thin blood smears were made on-site, fixed in methanol for 3 min, air-dried, and stored in slide boxes. At the KTTRS laboratory, the slides were stained in 10% Giemsa for 30 min at room temperature and examined using an Olympus cx22 light microscope at × 1000 magnification.

#### Mice inoculation

Where possible, infected dogs were resampled, or their blood in EDTA tubes was used to inoculate mice intraperitoneally with 0.5–1.0 ml of infected blood to detect subpatent *Trypanosoma* infections. The inoculated mice were labelled and monitored at KTTRS, with daily checks for parasitaemia using tail blood and Giemsa-stained smears.

### Off-site activities

#### Sample transportation and storage

Blood in EDTA and plain tubes were chilled on ice in a vehicle-operated thermo-electric cooler box and transported on the day of collection to KTTRS for processing and temporary storage. Blood in EDTA was directly stored at − 20 °C while blood in plain tubes was allowed to separate and clot overnight at room temperature. The resultant serum was transferred into 1 ml sterile vials and stored at − 20 °C. Serum and blood in EDTA tubes were stored at − 20 °C for about three weeks at each time point before being transported to the main laboratory in Lusaka (Zambia’s capital city). The frozen blood in EDTA tubes and serum samples were transported on ice in a vehicle-operated thermo-electric cooler box for molecular and serological diagnosis at the University of Zambia’s School of Veterinary Medicine.

#### Sample analysis

Blood in EDTA was thawed and used to extract genomic DNA at the University of Zambia, School of Veterinary Medicine’s trypanosomiasis laboratory. DNA was extracted using a mammalian blood DNA isolation kit (Roche Applied Science). About 200 μl of dog DNA per sample was eluted into sterile Eppendorf tubes and stored at − 80 °C until use. Serum samples were also stored at − 80 °C for future use.

#### Serodiagnosis

The ELISA assay followed a protocol described by Thuy et al.^61^ with some modifications. The recombinant GM6 antigen (rTeGM6) derived from *T. evansi* and the anti-dog IgG antibody was used to detect *Trypanosoma* antibodies in the extracted dog serum. Briefly, each well of microplates (Maxisorp, Nalgene-Nunc) was coated overnight with 50 µl (1 μg/ml) of the rTeGM6 antigen. The antigen-coated wells were washed once with phosphate-buffered saline (PBS) containing 0.05% Tween 20 (PBS-T) and incubated with PBS containing 5% skim milk at room temperature for 1 h. The wells were washed once with PBS-T and serum samples (50 µl/well) diluted at 250 times with PBS containing 5% skim milk were added and incubated at room temperature for 2 h. The wells were washed thrice with PBS-T before adding 100 µl per well of 10,000 times diluted Goat anti-Dog IgG antibody conjugated with horseradish peroxidase (BETHYL Laboratories). After 1 h of incubation at room temperature, the wells were washed thrice with PBS-T. Tetramethylbenzidine (100 µl) was added to each well and incubated for 30 min at room temperature. Finally, 100 µl stop solution (1 M sulphuric acid) and the absorbance read at 450 nm. After subtracting the blank well OD value, the negative samples’ standard deviation (SD) and average OD values were calculated. The cut-off ELISA values were calculated as mean ± 3SD of OD values using three negative control sera^48^. A confirmed *Trypanosoma*-positive dog from Mfuwe (Mambwe district) and three *Trypanosoma*-negative and non-tsetse exposed dogs from Lusaka were used as positive and negative controls, respectively.

#### ITS1 PCR

This study adopted a modified ITS1-PCR described by Gaithuma et al.^16^ to identify diverse *Trypanosoma* species. The PCR was performed in a final reaction volume of 10 µl containing; 2 µl of eluted DNA, 5 µl 2 × Ampdirect® Plus buffer, containing MgCl₂ and dNTPs (Shimadzu, Kyoto, Japan), 0.05 µl BioTaq HS (Bioline, UK), 0.2 µl 2% dimethyl sulphoxide (DMSO), 2.25 µl nuclease-free water, and 0.25 µl each of 10 µM AITS primers. Thermal cycling was performed at 95 °C for 10 min, followed by 40 cycles of 94 °C for 30 s, 57 °C for 1 min and 72 °C for 2 min, and final extension at 72 °C for 10 min. The PCR products were loaded onto 1.5% agarose gel containing GelRed nucleic acid stain (Biotium, Fremont, CA, USA), and products were visualized under ultraviolet (UV) light in a transilluminator. DNA from a Zambian HAT sample reported by Namangala et al.^50^ was used as a positive control and nuclease-free water as a negative control.

#### SRA PCR

Species-specific primers that amplify a 284 bp fragment of the serum resistance-associated (SRA) gene were used to confirm the human-infective *T. b. rhodesiense*^62^. The SRA PCR was run in a final reaction volume of 10 µl containing; 2 µl of eluted DNA, 5 µl 2 × Ampdirect® Plus buffer, containing MgCl₂ and dNTPs (Shimadzu, Kyoto, Japan), 0.05 µl BioTaq HS (Bioline, UK), 2.55 µl nuclease-free water, and 0.2 µl each of 10 µM SRA primers. Thermal cycling was performed at 95 °C for 10 min, followed by 40 cycles of 94 °C for 30 s, 60 °C for 1 min and 72 °C for 1 min, and final extension at 72 °C for 5 min. Amplicons were visualized in a UV transilluminator on 2% agarose gel stained with GelRed nucleic acid stain^19^.

#### Sequencing and phylogenetic analysis

Positive PCR products were purified using a Monofas Purification kit (GL Science) according to the manufacturer’s instructions. Sequencing of purified PCR products was performed by BigDye™ Terminator v3.1 Cycle Sequencing Kit (Applied Biosystems; Thermo Fisher Scientific, Foster City, CA). Purified sequence products were subjected to capillary electrophoresis using the ABI 3130 Genetic Analyzer (Applied Biosystems Japan Ltd., Tokyo, Japan). MEGA 7 was used to align sample sequences with several SRA reference sequences from Uganda (AF097331.1), Kenya (AJ345057.1), and Zambia (AJ345058.1, MN635738.1, MN635739.1, MN635740.1, MN635741.1, MN635742.1, and MN635743.1).

### Statistical analysis

Data were analysed using STATA 17.1 statistical software. Diagnostic results for microscopy were compared with those of GM6 ELISA and ITS1-PCR using the Kappa coefficient. PCR was used as the gold standard reference method to identify trypanosomes against GM6 ELISA as microscopy was not carried out on all dog samples. The sensitivity and specificity of GM6 ELISA were calculated as the number of ELISA positive and negative divided by the total number of PCR positives and negatives, respectively. Geneious ® 10.2.6 Bioinformatics software was used to edit and analyse raw DNA sequenced data (AB1 file extensions).

### Ethics

Ethical approval for this study was granted by the University of Zambia Biomedical Research Ethics Committee and the National Health Research Ethics Board under reference number 016-02-18. The study’s aims, procedures, and outcomes were explained to participants and assured confidentiality before initiating the study. All participants gave informed consent, and individuals below 18 years old accented after receiving parental or guardian consent. Further permission to conduct research within these chiefdoms was sought from traditional leaders and other relevant authorities. The care and maintenance of animals conformed with the ethical standards for animal use and welfare and ARRIVE guidelines (https://arriveguidelines.org/).

## Data Availability

Sequence data supporting our study findings have been deposited in the GenBank under accession numbers OL961811, OL961812, and OL961813. Other datasets generated during and/or analysed during the current study are available from the corresponding author upon reasonable request.

## References

[1] WHO. *Report of the First WHO Stakeholders Meeting on Rhodesiense Human African Trypanosomiasis, Geneva, 20–22 October 2014* (World Health Organisation, 2015).

[2] Morrison, L. J. & Macleod, A. African trypanosomiasis. *Parasite Immunol.* **33**, 421–422 (2011). 21609334 10.1111/j.1365-3024.2011.01302.x PMC3443387

[3] Kennedy, P. G. E. Clinical features, diagnosis, and treatment of human African trypanosomiasis (sleeping sickness). *Lancet Neurol.* **12**, 186–194 (2013). 23260189 10.1016/S1474-4422(12)70296-X

[4] Odeniran, P. O., Macleod, E. T., Ademola, I. O. & Welburn, S. C. Molecular identification of bloodmeal sources and trypanosomes in *Glossina* spp., *Tabanus* spp. and *Stomoxys* spp. trapped on cattle farm settlements in southwest Nigeria. *Med. Vet. Entomol.* **33**, 269–281 (2019). 30730048 10.1111/mve.12358

[5] Mwiinde, A. M. *et al.* Estimating the economic and social consequences for patients diagnosed with human African trypanosomiasis in Muchinga, Lusaka and Eastern Provinces of Zambia (2004–2014). *Infect. Dis. Poverty* **6**, 150 (2017). 29017597 10.1186/s40249-017-0363-6 PMC5634962

[6] Franco, J. R. *et al.* Monitoring the elimination of human African trypanosomiasis: Update to 2016. *PLoS Negl. Trop. Dis.* **12**, e0006890–e0006890 (2018). 30521525 10.1371/journal.pntd.0006890 PMC6283345

[7] Vourchakbé, J., Tiofack, Z. A. A., Kante, T. S., Mpoame, M. & Simo, G. Molecular identification of *Trypanosoma brucei gambiense* in naturally infected pigs, dogs and small ruminants confirms domestic animals as potential reservoirs for sleeping sickness in Chad. *Parasite* **27**, 63 (2020). 33206595 10.1051/parasite/2020061 PMC7673351

[8] Welburn, S. C. *et al.* Identification of human-infective trypanosomes in animal reservoir of sleeping sickness in Uganda by means of serum-resistance-associated (SRA) gene. *Lancet (London, England)* **358**, 2017–2019 (2001). 11755607 10.1016/S0140-6736(01)07096-9

[9] von Wissmann, B. *et al.* Factors associated with acquisition of human infective and animal infective trypanosome infections in domestic livestock in Western Kenya. *PLoS Negl. Trop. Dis.* **5**, e941 (2011). 21311575 10.1371/journal.pntd.0000941 PMC3022529

[10] Munangandu, H. M., Siamudaala, V., Munyeme, M. & Nalubamba, K. S. A review of ecological factors associated with the epidemiology of wildlife trypanosomiasis in the luangwa and zambezi valley ecosystems of zambia. *Interdiscip. Perspect. Infect. Dis.* **2012**, 372523 (2012). 22693499 10.1155/2012/372523 PMC3368204

[11] Hamill, L. C., Kaare, M. T., Welburn, S. C. & Picozzi, K. Domestic pigs as potential reservoirs of human and animal trypanosomiasis in Northern Tanzania. *Parasit. Vectors* **6**, 322 (2013). 24499540 10.1186/1756-3305-6-322 PMC3843548

[12] Matovu, E. *et al.* Haemoparasitic infections in cattle from a *Trypanosoma brucei rhodesiense* sleeping sickness endemic district of Eastern Uganda. *Trop. Med. Infect. Dis.* **5**, 24 (2020). 32046044 10.3390/tropicalmed5010024 PMC7157584

[13] Buyst, H. The epidemiology of sleeping sickness in the historical Luangwa valley. *Ann. Soc. Belg. Med. Trop.* **1920**(57), 349–359 (1977). 345980

[14] Kinghorn, A., Yorke, W. & Lloyd, L. Final report of the luangwa sleeping sickness commission of the British South Africa Company 1911–1912. *Ann. Trop. Med. Parasitol.* **7**, 183–302 (1913). 10.1080/00034983.1913.11687609

[15] Woolhouse, M. E. J., Bealby, K., McNamara, J. J. & Silutongwe, J. Trypanosome infections of the tsetse fly *Glossina pallidipes* in the Luangwa Valley, Zambia. *Int. J. Parasitol.* **24**, 987–993 (1994). 7772128 10.1016/0020-7519(94)90164-3

[16] Gaithuma, A. K. *et al.* A single test approach for accurate and sensitive detection and taxonomic characterization of trypanosomes by comprehensive analysis of internal transcribed spacer 1 amplicons. *PLoS Negl. Trop. Dis.* **13**, 1–20 (2019). 10.1371/journal.pntd.0006842 PMC6414030 30802245

[17] Dillmann, J. S. & Townsend, A. J. A trypanosomiasis survey of wild animals in the Luangwa Valley, Zambia. *Acta Trop.* **36**, 349–356 (1979). 44099

[18] Anderson, N. E. *et al.* Characterisation of the wildlife reservoir community for human and animal trypanosomiasis in the Luangwa Valley, Zambia. *PLoS Negl. Trop. Dis.* **5**, e1211 (2011). 21713019 10.1371/journal.pntd.0001211 PMC3119639

[19] Squarre, D. *et al.* Diversity of trypanosomes in wildlife of the Kafue ecosystem, Zambia. *Int. J. Parasitol. Parasites Wildl.* **12**, 34–41 (2020). 32420023 10.1016/j.ijppaw.2020.04.005 PMC7215119

[20] Simukoko, H. *et al.* The comparative role of cattle, goats and pigs in the epidemiology of livestock trypanosomiasis on the plateau of eastern Zambia. *Vet. Parasitol.* **147**, 231–238 (2007). 17493757 10.1016/j.vetpar.2007.04.005 PMC2771273

[21] Laohasinnarong, D. *et al.* Studies of trypanosomiasis in the Luangwa valley, north-eastern Zambia. *Parasit. Vectors* **8**, 497 (2015). 26419347 10.1186/s13071-015-1112-y PMC4589067

[22] Songa, E. B. *et al.* Evidence for widespread asymptomatic *Trypanosoma rhodesiense* human infection in the Luangwa Valley (Zambia). *Trop. Med. Parasitol.* **42**, 389–393 (1991). 1796239

[23] Dukes, P., Scott, C. M., Rickman, L. R. & Wupara, F. Sleeping sickness in the Luangwa Valley of Zambia. A preliminary report of the 1982 outbreak at Kasyasya village. *Bull. Soc. Pathol. Exot. Filiales* **76**, 605–613 (1983). 6673853

[24] Mwanakasale, V., Songolo, P., Babaniyi, O. & Simarro, P. Clinical presentation of human African trypanosomiasis in Zambia is linked to the existence of strains of *Trypanosoma brucei rhodesiense* with varied virulence: Two case reports. *J. Med. Case Rep.* **8**, 2–4 (2014). 24529084 10.1186/1752-1947-8-53 PMC3930019

[25] Helleberg, B. B., Gudmundsson, K. S. S., Kurtzhals, J. A. L. & Helleberg, M. Second stage human African Trypanosomiasis with *Trypanosoma brucei rhodesiense* treated with fexinidazole. *Lancet Infect. Dis.* **23**, e505 (2023). 37890908 10.1016/S1473-3099(23)00358-4

[26] Okiwelu, S. N. Host preference and trypanosome infection rates of *Glossina morsitans morsitans* Westwood in the Republic of Zambia. *Ann. Trop. Med. Parasitol.* **71**, 101–107 (1977). 849014 10.1080/00034983.1977.11687166

[27] Newberry, K., Boreham, P. F. L. & Sheppard, M. A preliminary investigation of the effect of age, sex and time of collection on the feeding patterns of Glossina morsitans morsitans Westw. in Zambia. *Trans. R. Soc. Trop. Med. Hyg.* **76**, 79–82 (1982). 7200644 10.1016/0035-9203(82)90024-4

[28] Van Den Bossche, P. & Staak, C. The importance of cattle as a food source for *Glossina morsitans morsitans* in Katete district, Eastern Province, Zambia. *Acta Trop.* **65**, 105–109 (1997). 9164604 10.1016/S0001-706X(97)00658-X

[29] Konnai, S. *et al.* Detection of *Trypanosoma brucei* in field-captured tsetse flies and identification of host species fed on by the infected flies. *Vector Borne Zoonotic Dis.* **8**, 565–573 (2008). 18399780 10.1089/vbz.2007.0223

[30] Gaithuma, A. *et al.* Blood meal sources and bacterial microbiome diversity in wild-caught tsetse flies. *Sci. Rep.* **10**, 5005 (2020). 32193415 10.1038/s41598-020-61817-2 PMC7081217

[31] Van Den Bossche, P., Doran, M. & Connor, R. J. An analysis of trypanocidal drug use in the Eastern Province of Zambia. *Acta Trop.* **75**, 247–258 (2000). 10708665 10.1016/S0001-706X(00)00059-0

[32] Sinyangwe, L. *et al.* Trypanocidal drug resistance in eastern province of Zambia. *Vet. Parasitol.* **119**, 125–135 (2004). 14746972 10.1016/j.vetpar.2003.11.007

[33] Delespaux, V., Dinka, H., Masumu, J., Van den Bossche, P. & Geerts, S. Five-fold increase in *Trypanosoma congolense* isolates resistant to diminazene aceturate over a seven-year period in Eastern Zambia. *Drug Resist. Updat.* **11**, 205–209 (2008). 18993109 10.1016/j.drup.2008.10.002

[34] Chitanga, S. *et al.* High prevalence of drug resistance in animal trypanosomes without a history of drug exposure. *PLoS Negl. Trop. Dis.* **5**, e1454 (2011). 22206039 10.1371/journal.pntd.0001454 PMC3243716

[35] Dennis, J. W. *et al.**Sodalis glossinidius* prevalence and trypanosome presence in tsetse from Luambe National Park, Zambia. *Parasites Vectors* **7**, 1–11 (2014). 25138709 10.1186/1756-3305-7-378 PMC4153904

[36] Nakamura, Y. *et al.* Genetic diversity and population structure of *Glossina morsitans morsitans* in the active foci of human African trypanosomiasis in Zambia and Malawi. *PLoS Negl. Trop. Dis.* **13**, e0007568 (2019). 31344039 10.1371/journal.pntd.0007568 PMC6657825

[37] Mulenga, G. *et al.**Challenges in the Diagnostic Performance of Parasitological and Molecular Tests in the Detection of African Trypanosomiasis in Cattle in Mambwe District in Eastern Zambia* (2020). 10.3390/tropicalmed6020068 PMC8167722 33946506

[38] Namangala, B. *et al.* Detection of human-infective trypanosomes in acutely-infected Jack Russel from Zambia’s south Luangwa national park by loop-mediated isothermal amplification. *Tanzan. Vet. J.* **28**, 12–20 (2013).

[39] Lisulo, M. *et al.* Determination of the prevalence of African trypanosome species in indigenous dogs of Mambwe district, eastern Zambia, by loop-mediated isothermal amplification. *Parasit. Vectors* **7**, 19 (2014). 24411022 10.1186/1756-3305-7-19 PMC3895695

[40] Lisulo, M. *et al.* Dogs’ health and demographics in wildlife-populated and tsetse-infested villages of Mambwe district, eastern Zambia. *Prev. Vet. Med.* **217**, 105969 (2023). 37406502 10.1016/j.prevetmed.2023.105969

[41] Namangala, B. *et al.* Short report: Preliminary investigation of trypanosomosis in exotic dog breeds from Zambia’s Luangwa and Zambezi Valleys using LAMP. *Am. J. Trop. Med. Hyg.* **89**, 116–118 (2013). 23716412 10.4269/ajtmh.13-0078 PMC3748466

[42] Moloo, S. K., Losos, G. J. & Kutuza, S. B. Transmission of *Trypanosoma brucei* to cats and dogs by feeding of infected goats. *Ann. Trop. Med. Parasitol.* **67**, 331–334 (1973). 4761942 10.1080/00034983.1973.11686894

[43] Raina, A. K., Kumar, R., Sridhar, V. S. R. & Singh, R. P. Oral transmission of *Trypanosoma evansi* infection in dogs and mice. *Vet. Parasitol.* **18**, 67–69 (1985). 4049728 10.1016/0304-4017(85)90009-3

[44] Pillay, D. *et al.**Trypanosoma vivax* GM6 antigen: A candidate antigen for diagnosis of African animal trypanosomosis in cattle. *PLoS One* **8**, 1–10 (2013). 10.1371/journal.pone.0078565 PMC3808341 24205263

[45] Nguyen, T.-T. *et al.* Application of crude and recombinant ELISAs and immunochromatographic test for serodiagnosis of animal trypanosomosis in the Umkhanyakude district of KwaZulu-Natal province, South Africa. *J. Vet. Med. Sci.* 14–330 (2014). 10.1292/jvms.14-0330 PMC4363025 25342634

[46] Mossaad, E. *et al.* Utilization of crude and recombinant ELISAs for serodiagnosis of camel trypanosomosis in Sudan. *Vet. Parasitol. Reg. Stud. Rep.* **16**, 100278 (2019). 10.1016/j.vprsr.2019.100278 31027599

[47] Elata, A. *et al.* Serological and molecular detection of selected hemoprotozoan parasites in donkeys in West Omdurman, Khartoum State, Sudan. *J. Vet. Med. Sci.* **82**, 286–293 (2020). 31969541 10.1292/jvms.19-0534 PMC7118482

[48] Davaasuren, B. *et al.* The evaluation of GM6-based ELISA and ICT as diagnostic methods on a Mongolian farm with an outbreak of non-tsetse transmitted horse trypanosomosis. *Vet. Parasitol.* **244**, 123–128 (2017). 28917303 10.1016/j.vetpar.2017.07.036

[49] Nguyen, T. T. *et al.* Diagnostic value of the recombinant tandem repeat antigen TeGM6-4r for surra in water buffaloes. *Vet. Parasitol.* **201**, 18–23 (2014). 24524896 10.1016/j.vetpar.2014.01.009

[50] Namangala, B. *et al.* The use of Loop-mediated Isothermal Amplification (LAMP) to detect the re-emerging Human African Trypanosomiasis (HAT) in the Luangwa and Zambezi valleys. *Parasites Vectors* **5**, 1–5 (2012). 23211002 10.1186/1756-3305-5-282 PMC3533913

[51] Squarre, D. *et al.* Human African trypanosomiasis in the Kafue National Park, Zambia. *PLoS Negl. Trop. Dis.* **10**, e0004567 (2016). 27196336 10.1371/journal.pntd.0004567 PMC4873190

[52] Kaggwa, E., Munyua, W. K. & Mugera, G. M. Relapses in dogs experimentally infected with *Trypanosoma brucei* and treated with diminazene aceturate or isometamidium chloride. *Vet. Parasitol.* **27**, 199–208 (1988). 3369073 10.1016/0304-4017(88)90034-9

[53] Nwosu, C. O. & Ikeme, M. M. Parasitaemia and clinical manifestations in *Trypanosoma brucei* infected dogs. *Rev. Elev. Med. Vet. Pays Trop.* **45**, 273–277 (1992). 1339994 10.19182/remvt.8916

[54] Matete, G. O. Occurrence, clinical manifestation and the epidemiological implications of naturally occurring canine trypanosomosis in western Kenya. *Onderstepoort J. Vet. Res.* **70**, 317–323 (2003). 14971734 10.4102/ojvr.v70i4.296

[55] Abenga, J., David, K., Ezebuiro, C. & Lawani, F. Observations on the tolerance of young dogs (puppies) to infection with *Trypanosoma congolense*. *Afr. J. Clin. Exp. Microbiol.* **6**, 33 (2005).

[56] Ezeokonkwo, R. C. *et al.* Comparative haematological study of single and mixed infections of mongrel dogs with *Trypanosoma congolense* and *Trypanosoma brucei brucei*. *Vet. Parasitol.* **173**, 48–54 (2010). 20638796 10.1016/j.vetpar.2010.06.020

[57] Nwoha, I. O. A review on trypanosomosis in dogs and cats. *Afr. J. Biotechnol.* **12**, 6432–6442 (2013). 10.5897/AJB2013.12093

[58] Bwalya Umar, B. & Kapembwa, J. Economic benefits, local participation, and conservation ethic in a game management area: Evidence from Mambwe, Zambia. *Trop. Conserv. Sci.* **13**, 1940082920971754 (2020). 10.1177/1940082920971754

[59] Nalubamba, K. S. *et al.* A study of naturally acquired canine babesiosis caused by single and mixed babesia species in Zambia: Clinicopathological findings and case management. *J. Parasitol. Res.* **2015**, 985015 (2015). 26682062 10.1155/2015/985015 PMC4670660

[60] Desquesnes, M. *et al.* Compendium of standard diagnostic protocols for animal trypanosomoses of African origin (OIE Reference Laboratory for Animal Trypanosomoses of African origin). (2016).

[61] Thuy, N. T., Goto, Y., Lun, Z. R., Kawazu, S. I. & Inoue, N. Tandem repeat protein as potential diagnostic antigen for *Trypanosoma evansi* infection. *Parasitol. Res.* **110**, 733–739 (2012). 21927872 10.1007/s00436-011-2632-9

[62] Radwanska, M. *et al.* The serum resistance-associated gene as a diagnostic tool for the detection of *Trypanosoma brucei rhodesiense*. *Am. J. Trop. Med. Hyg.* **67**, 684–690 (2002). 12518862 10.4269/ajtmh.2002.67.684

